# One-year outcomes of microshunt implantation in pseudoexfoliation glaucoma

**DOI:** 10.1371/journal.pone.0256670

**Published:** 2021-08-27

**Authors:** Matthias Nobl, Sigrid Freissinger, Stefan Kassumeh, Siegfried Priglinger, Marc J. Mackert

**Affiliations:** Department of Ophthalmology, Ludwig-Maximilians-University Munich, Munich, Germany; Cairo University Kasr Alainy Faculty of Medicine, EGYPT

## Abstract

**Purpose:**

To compare the safety and efficacy of microshunt implantation augmented with Mitomycin C in patients with pseudoexfoliation glaucoma (PEXG) and primary open-angle glaucoma (POAG).

**Methods:**

In this retrospective, single centre, interventional study, 46 eyes of 41 patients with PEXG (20 eyes) and POAG (26 eyes) underwent microshunt implantation. Definition of failure was an intraocular pressure (IOP) lower than 5 or higher than 17mmHg on two consecutive visits, an IOP reduction lower than 20% on two consecutive visits, the need of surgical revisions or reoperations or loss of light perception. Outcome was rated as complete success if achieved without medication, otherwise as qualified success. Furthermore, postoperative complications and interventions were compared between the two groups.

**Results:**

Patient demographics were similar, except for older age in the PEXG group (70.9±8.6 versus 77.6±8; *p* = 0.02). Mean IOP dropped from 21.5±5.8mmHg (PEXG) and 18.2±4.5mmHg (POAG) at baseline to 12.8±3.0mmHg (*p*<0.0001) and 12.9±4.2mmHg (*p*<0.0001), respectively, at one year. Mean number of medications were reduced from 2.8±1.3 to 0.3±0.8 for PEXG patients (*p*<0.0001) and from 2.7±1.3 to 0.3±0.8 for POAG patients (*p*<0.0001). At one year of follow-up 75.0% of PEXG patients achieved complete success and 80.0% qualified success. In the POAG group rates were 73.1% and 76.9%, respectively. Postoperative complications were comparable between both groups, except for higher rates of hypotony (*p* = 0.04) and choroidal detachment (*p* = 0.03) in the PEXG group.

**Conclusion:**

Microshunt implantation demonstrated similar efficacy results in PEXG and POAG eyes at a follow-up of 12 months. Higher rates of transient hypotony and choroidal detachment were observed in PEXG eyes.

## Introduction

Glaucoma is a common disease and one of the leading causes of blindness worldwide. The number of persons affected by glaucoma is predestined to rise significantly over the next years mostly due to the aging population [1]. Treatment of glaucoma is usually started using topical pharmaceutical therapy or laser trabeculoplasty. Nevertheless, in patients showing advanced visual field defects, insufficiently controlled intraocular pressure (IOP) despite medical treatment or side effects of eye drop therapy, filtering surgery is often required to stop or slow the disease progression.

Pseudoexfoliation glaucoma (PEXG) shows specific alterations within the anterior segment and is the most common identifiable cause of open angle glaucoma [2,3]. In contrast to primary open angle glaucoma (POAG), patients suffering from PEXG are often older, show a higher IOP and more advanced visual field defects at initial presentation [4]. In addition, PEXG shows a higher short-term fluctuation in IOP [5]. Taking these findings into account PEXG seems to be more difficult to manage [4]. It is known that PEXG shows a more rapidly progressive course and that the topical medical treatment is more likely to fail in slowing this progression in the long-term [6,7]. Therefore, filtering surgery in PEXG is necessary more often and at an earlier time. Surgical options in PEXG are the same as in POAG. However, surgical success rates regarding trabeculectomy have been suggested to be significantly lower in PEXG compared to POAG [8]. It was speculated that surgery might exacerbate preexisting blood aqueous barrier breakdown in PEXG, leading to more severe postoperative inflammation and thus accelerating bleb scarring and worsening of bleb function [8,9].

The MicroShunt (Santen, Osaka, Japan) is a new minimally invasive glaucoma surgery device. It is made of a polymer called poly(styrene-block-isobutylene-block-styrene) or SIBS [10]. Critical features of SIBS are the softness, flexibility and biocompatibility [10]. As a result, histological evaluation after ocular implantation of SIBS drainage tubes in rabbits showed reduced collagen deposition around the tubes and the absence of myofibroblasts [10]. Previously published studies showed high rates of qualified and complete success with few complications for microshunt implantation as a standalone procedure or combined with cataract surgery in POAG [11–13].

The purpose of this study is to confirm the safety and efficacy of microshunt implantation in patients with uncontrolled PEXG in comparison to POAG. It is to the best of our knowledge the first study designed to evaluate the microshunt in PEXG.

## Methods

### Study design

This is an investigator initiated, single centre, retrospective, interventional study with a concurrent comparison group of consecutive patients receiving a microshunt with Mitomycin C (MMC) from June 2019 to February 2020 as a standalone procedure or in combination with cataract surgery. The device was implanted in eyes with an IOP above target pressure or with progression on the maximum tolerated glaucoma medication. The study adhered to the tenets of the Declaration of Helsinki and institutional review board approval (ID 17–529) was obtained. Informed consent was obtained from all patients prior to surgery.

### Baseline measurements

For all patients baseline measurements were performed at the time the decision for surgery was made. Data collected included: age, sex, eye, glaucoma diagnosis, glaucoma treatment, best-corrected visual acuity, IOP using Goldmann applanation tonometry, lens status, central corneal thickness and history of prior surgery or laser treatment. In addition, standard automated perimetry was performed before patients underwent surgery.

### Inclusion and exclusion criteria

All patient receiving a subconjunctival microshunt at our clinic from June 2019 onwards were eligible for the study. Inclusion criterion was diagnosis of POAG or PEXG. Patients had to be older than 18 years at the time of surgery to be included into the study.

Exclusion criteria were diagnosis of closed angle glaucoma, neovascular glaucoma or uveitic glaucoma. Patients were also excluded if they had a follow-up less than 8 months postoperatively. Prior surgery, also prior incisional filtering glaucoma surgery was not an exclusion criterion.

### Preoperative management

Ten days prior to surgery all antiglaucomatous eye drops were stopped on the affected eye. Instead, orally carbonic anhydrase inhibitor as well as topical preservative free dexamethasone eye drops were prescribed three times a day, to achieve a situation of best possible conjunctival status to optimize surgical conditions and outcome. The antiglaucomatous therapy of the partner eye was left unchanged.

### Implantation

Microshunt implantation was performed as a standalone procedure or in combination with cataract surgery by two experienced surgeons (M.M. and S.P.) in an in-patient setting. Under general anaesthesia or retrobulbar block, a corneal traction suture was placed. The surgeon examined the exposed superior conjunctiva to decide on the optimal device placement. A small incision of the conjunctiva and the Tenon’s capsule was made at the limbus in the superior temporal or superior nasal quadrant. Via blunt preparation with scissors, Tenon’s layer was carefully dissected from the underlying episcleral one. Bipolar diathermy was applied when necessary and episcleral tissue was removed using a hockey knife, leaving a clean surgical field. Three 0.2mg/ml MMC soaked sponges were applied in the flap under Tenon’s layer of the flap for two minutes. After sponge removal, the eye was carefully rinsed with balanced salt solution. The included template was used to mark a point 3 mm posterior to the limbus. There a small, triangular scleral pocket was made using a 1mm blade. From the apex of the scleral pocket a tunnel was created into the anterior chamber employing a 25 G needle. Care was taken that the end of the tunnel was just above the iris. A paracentesis was performed. With a forceps the microshunt was inserted into the scleral tunnel and advanced into the anterior chamber so that the fins of the device become adequately wedged in the scleral pocket. The proximal end of the shunt extending 2–3 mm into the anterior chamber was inspected whether it was oriented bevel up and if it neither touched the cornea nor the iris. At the distal end of the tube flow of aqueous humor was confirmed by observation of drop formation. Tenon’s layer and conjunctiva were carefully closed over the implant using 9–0 vicryl sutures ensuring that the implant lies flush against the sclera and was not caught in Tenon’s layer. After the peribulbar injection of 4mg dexamethasone and the instillation of dexa-gentamicin eye ointment an eye patch was applied.

### Postoperative management

Following surgery, the treatment with oral carbonic anhydrase inhibitor and topical preservative free dexamethasone eye drops was terminated. Topical antibiotics were used four times a day for one week, whereas steroid eyedrops were administered hourly for one week and then slowly reduced over the course of the following 2 months, depending on the grade of postoperative inflammation. Steroid ointment at night was used for 4 weeks.

In an in-patient setting patients were seen daily till discharge, usually 2–5 days postoperatively. After a first out-patient check-up about one week postoperatively, further appointments were made at the surgeon’s discretion. All visits included an in-depth ocular examination of the bleb and the anterior segment as well as Goldmann applanation tonometry. Funduscopy was performed without pupillary dilatation. Needling, anterior chamber reformation using viscoelastics, surgical revision and re-introduction of glaucoma therapy were performed as decided by the surgeon.

Needling was performed in the operation theatre under a surgical microscope to allow best possible visualization. After topical anaesthesia, lidocaine was injected under the conjunctiva in proximity of the bleb. A 27 G needle was used to relieve adhesions in the subconjunctival space above and below the device, performing careful, sweeping motions. At the end 0.1 ml of 5-Fluoruracil (5-FU) was injected subconjunctivally next to the bleb.

### Surgical revision

Surgical revision was performed in an in-patient setting, if the IOP was elevated with a flat and scarred bleb. Under retrobulbar block a corneal traction suture was placed. The conjunctiva was carefully dissected and the scar tissue surrounding the implant was removed. MMC soaked sponges were applied for two minutes. After sponge removal, the eye was carefully rinsed with balanced salt solution. Flow through the implant and therefore good function was confirmed by observation of drop formation at the distal end of the implant. Conjunctiva was closed with 9–0 vicryl, ensuring a watertight seal. After the peribulbar injection of 4mg dexamethasone and the instillation of dexa-gentamicin eye ointment an eye patch was applied. Postoperative treatment regime was identical to the treatment after initial implantation.

### Outcome measurements

Primary outcome was percentage of patients achieving complete success at 12 months of follow-up. With consideration of the World Glaucoma Association’s Guidelines on Design and Reporting of Glaucoma Surgical Trials failure was defined as any of the following: (1) IOP of more than 17mmHg or less than 5mmHg on two consecutive visits, (2) IOP reduction of less than 20% from baseline IOP on two consecutive visits, (3) surgical revision or reoperation or (4) loss of light perception. Office visits took place according to our postoperative follow-up regimen. After the operation, patients routinely stayed in hospital for two days. Further outpatient visits were scheduled one to four weeks, four to eight weeks, three to four months, four to six months and eight to fourteen months postoperative. The first and second postoperative day were not considered consecutive visits. So, for example, if a patient had IOP readings of under 5mmHg on the first two days postoperative but an IOP reading in the range of 5 to 17mmHg at the next visit, it was not considered as failure. Regarding failure criterion 3, needling was not considered a failure, whereas surgical revision with opening of the conjunctiva and excision of scar tissue was. Missing of all failure criteria at one year of follow-up achieved without any antiglaucomatous medication was rated as complete success. Otherwise, with any antiglaucomatous medication it was rated as qualified success.

Postoperative interventions and complications were recorded. Postoperative interventions included subconjunctival injection of 5-FU, needling procedures and anterior chamber reformation using an intracameral viscoelastics. Postoperative complications that were noted included hyphema, corneal erosion, corneal dellen, corneal edema, leak or dehiscence with positive Seidel test, hypotony, flat anterior chamber, choroidal detachment, macular folds, exposed implant and blebitis.

Cases that required an additional glaucoma surgery were only included in the postoperative analysis up to the moment of the decision to intervene.

### Statistical analysis

Data were presented as mean and standard deviation, if not otherwise indicated. Percentages were calculated in categorial values to achieve better comparators between groups. Snellen visual acuity was converted to logMAR units. Statistical analysis and graph plotting was performed using GraphPad Prism 9 (GraphPad Software, San Diego, CA, USA). To calculate differences between two groups regarding ordinal scaled data Fisher’s exact test was used. To compare interval scaled data between two groups non-parametric Mann-Whitney-U Test was performed, as data was not normally distributed. Comparison of more than two groups was conducted using repeated measures ANOVA with Geisser-Greenhouse correction. Probability of success was assessed using Kaplan-Meier survival curves based on the aforementioned definitions of success. *P* < 0.05 was considered statistically significant.

## Results

### Study population

This study identified 46 eyes of 41 patients undergoing surgery with subconjunctival microshunt implantation using MMC from June 2019 to February 2020. Twenty-six eyes of 23 patients with POAG and 20 eyes of 18 patients with PEXG received microshunt implantation. The main baseline clinical and demographic characteristics are presented in Table 1. Patients in the POAG group (70.9 ± 8.6 years) were significantly younger than patients in the PEXG group (77.6 ± 8.1 years, *p* = 0.02). Baseline IOP was 18.2 ± 4.5mmHg in the POAG group and 21.4 ± 5.8mmHg in the PEXG group showing a significant difference (*p* = 0.04). Glaucoma severity, according to visual field mean deviation, was evenly distributed across the POAG group and the PEXG group. At the time of surgery 11 eyes (42.3%) were already pseudophakic in the POAG group versus 15 (75.0%) in the PEXG group. Combined surgery was performed on 4 eyes (15.4%) in the POAG groups versus 1 eye (5.0%) in the PEXG group. A total of 6 patients had previous glaucoma surgery. In the POAG group 2 had cyclophotocoagulation and 2 trabeculotomy. In the PEXG group 1 had cyclophotocoagulation and 1 trabeculectomy. Mean follow-up was 12.2 ± 1.6 months in the POAG group and 11.1 ± 2.4 months in the PEXG group (*p* = 0.13).

**Table 1:** Demographic and clinical data.

	POAG (n = 26)	PEXG (n = 20)	*p*
Age (y, mean ± SD)	70.9 ± 8.6	77.6 ± 8.1	0.02
Female [n (%)]	10 (43.5)	6 (33.3)	0.54
Pseudophakic [n (%)]	11 (42.3)	15 (75.0)	-
Bilateral cases [n (%)]	3 (11.5)	2 (10.0)	-
Combined surgery [n (%)]	4 (15.4)	1 (5.0)	-
CCT (μm, mean ± SD)	542 ± 33	543 ± 54	0.72
Baseline IOP (mmHg)	18.2 ± 4.5	21.4 ± 5.8	0.04
Baseline MD (mean ± SD)	-8.97 ± 7.12	-7.65 ± 5.59	0.37
Baseline BVCA (logmar, mean ± SD)	0.23 ± 0.19	0.18 ± 0.23	0.20
Baseline Medications (n, mean ± SD)	2.7 ± 1.3	2.8 ± 1.3	0.79

BCVA = best corrected visual acuity; CCT = central corneal thickness; IOP = intraocular pressure; MD = mean deviation; n = number, *p* = p-value; PEXG = pseudoexfoliation glaucoma; POAG = primary open angle glaucoma; SD = standard deviation, y = years.

### Intraocular pressure

**Fig 1:**
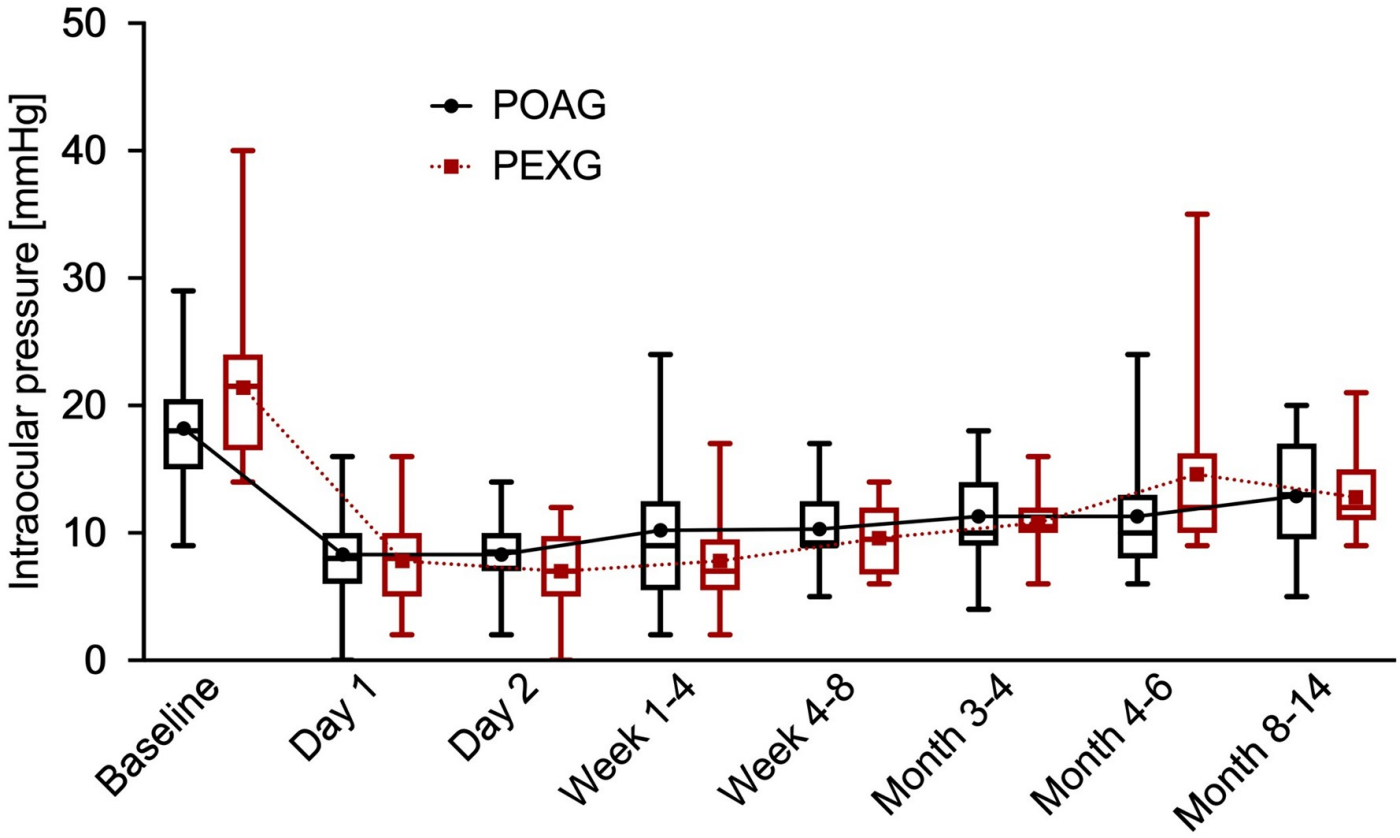
Intraocular pressure. Box and whisker plots of intraocular pressure preoperative and at each time of follow up for primary open angle glaucoma (POAG) and pseudoexfoliation glaucoma (PEXG). The middle lines indicate the median. The lower and upper ends of boxes indicate the lower and upper quartiles. Whiskers span from the absolute minimum to the absolute maximum. Additionally, mean values of intraocular pressure are shown, connected with a line.

**Fig 2:**
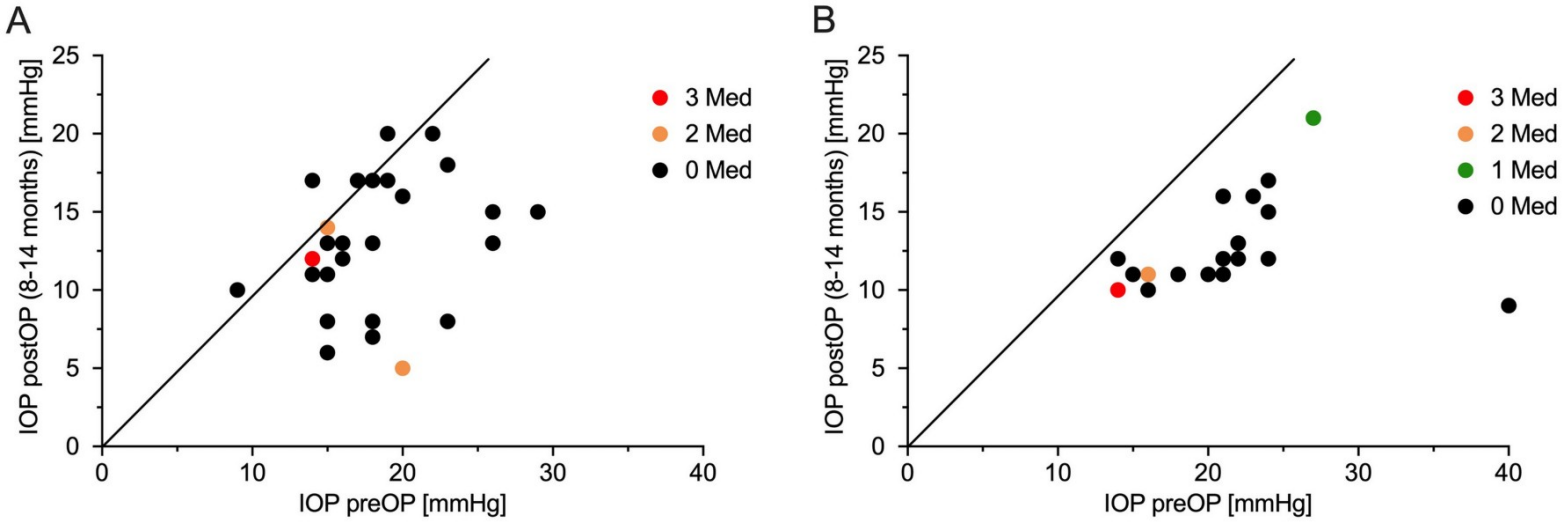
Scatter plot of intraocular pressure preoperatively versus one year postoperatively. (A) Primary open angle glaucoma; (B) Pseudoexfoliation glaucoma. Plotted according to number of antiglaucomatous medication at one year postoperative. IOP = intraocular pressure; preOP = preoperative; postOP = postoperative; Med = antiglaucomatous medication.

Mean IOP in the POAG group dropped from 18.2 ± 4.5mmHg at baseline to 12.9 ± 4.2mmHg (reduction of 5.3mmHg = 29.1%) at 12 months postoperatively. In the PEXG group mean IOP decreased from 21.4 ± 5.8mmHg at baseline to 12.8 ± 3.0 (reduction of 8.6mmHg = 40.2%) at 12 months of follow-up. The mean IOP reduction was statistically significant in the POAG group (*p* < 0.0001) as well as in the PEXG group (*p* < 0.0001). The magnitude of pressure reduction was significantly higher in the PEXG group than in the POAG group (*p* = 0.0473). During the entire follow-up no statistically significant difference was found regarding mean IOP at any given dates between the two groups. Fig 1 presents the changes in mean IOP in both groups across the follow-up of one year. The IOP readings pre- and postoperative after one year in relation to the number of antiglaucomatous medication are shown as scatter plot in Fig 2.

### Medical therapy

**Fig 3:**
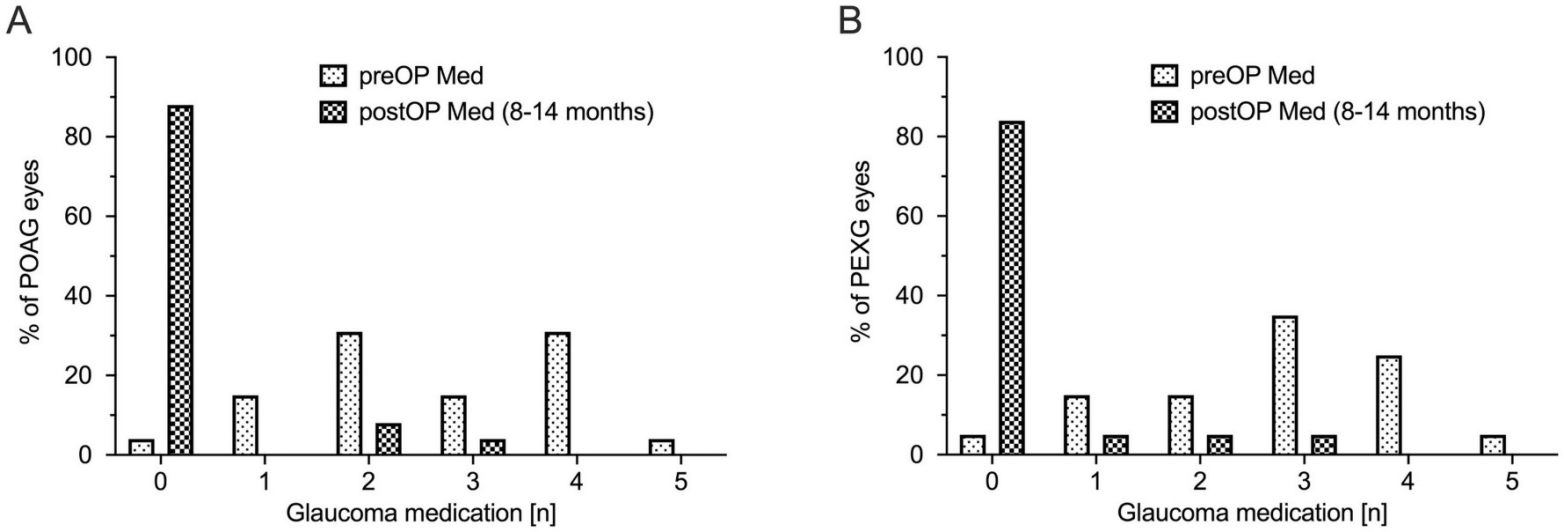
Number of antiglaucomatous medication. Number of antiglaucomatous medication preoperatively and one year postoperatively. (A) POAG = primary open angle glaucoma; (B) = PEXG = pseudoexfoliation glaucoma PreOP = preoperative intraocular pressure; postOP = postoperative intraocular pressure.

**Fig 4:**
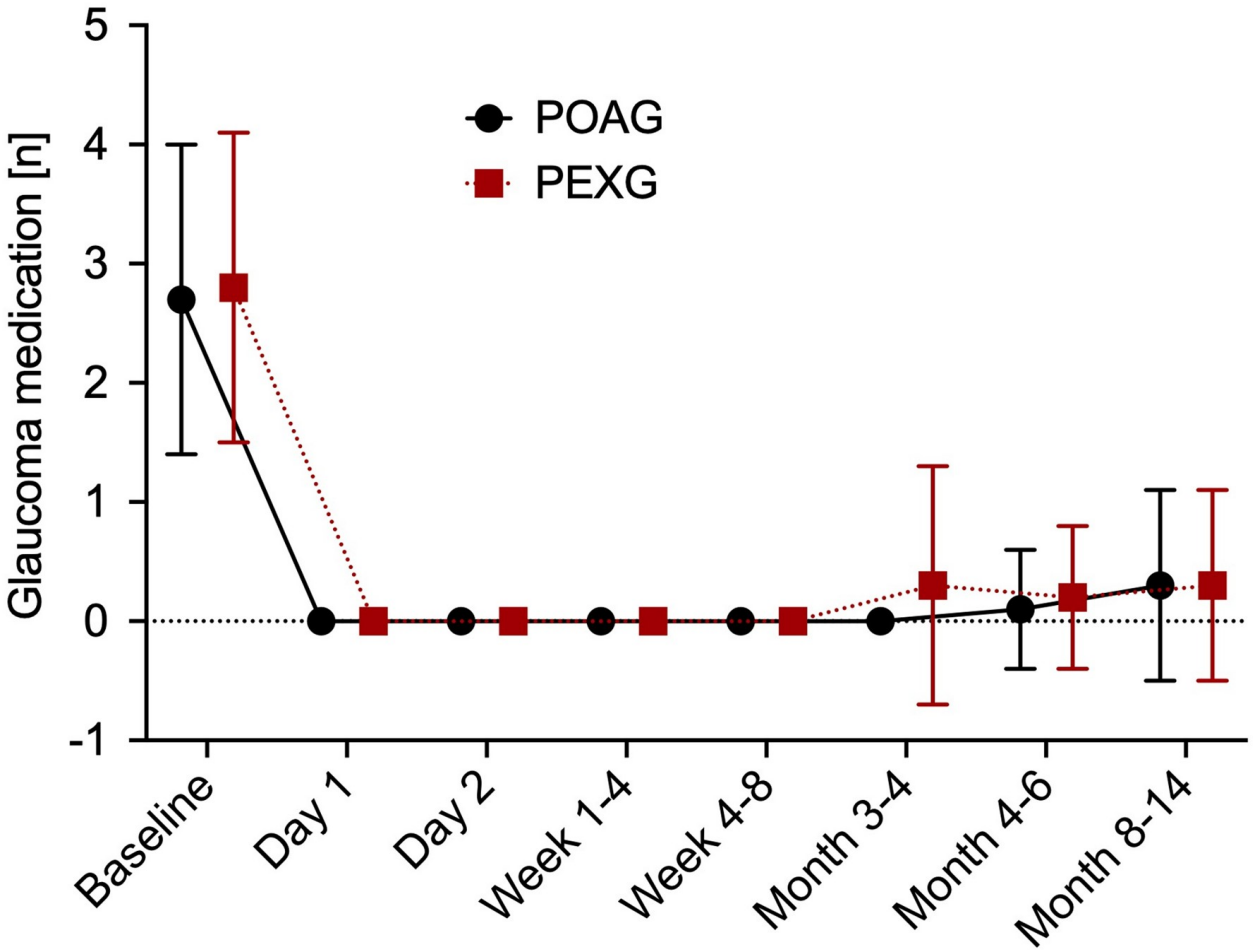
Medical therapy. Mean and standard deviation of number of antiglaucomatous medication at each time of follow-up for primary open angle glaucoma (POAG) and pseudoexfoliation glaucoma (PEXG).

A significant reduction of medications necessary to control IOP was seen in both groups. Mean number of medications in the POAG group dropped from 2.7 ± 1.3 at baseline to 0.3 ± 0.8 at 12 months postoperative (*p* < 0.0001). In the PEXG group mean number of medications decreased from 2.8 ± 1.3 at baseline to 0.3 ± 0.8 at 12 months of follow-up (*p* < 0.0001). There was no statistically significant difference regarding reduction of mean number of medications between the two groups (*p* = 0.95). As demonstrated in Fig 3, 6 eyes of 6 patients (13.3%) required medication to control IOP at one year of follow-up. In 3 patients of the POAG group (11.5%) as well as in 3 patients in the PEXG group (18.8%) therapy had to be restarted (*p* = 0.69). Fig 4 shows the changes in mean number of antiglaucomatous therapy in both groups across the follow-up of one year.

### Success rates

**Fig 5:**
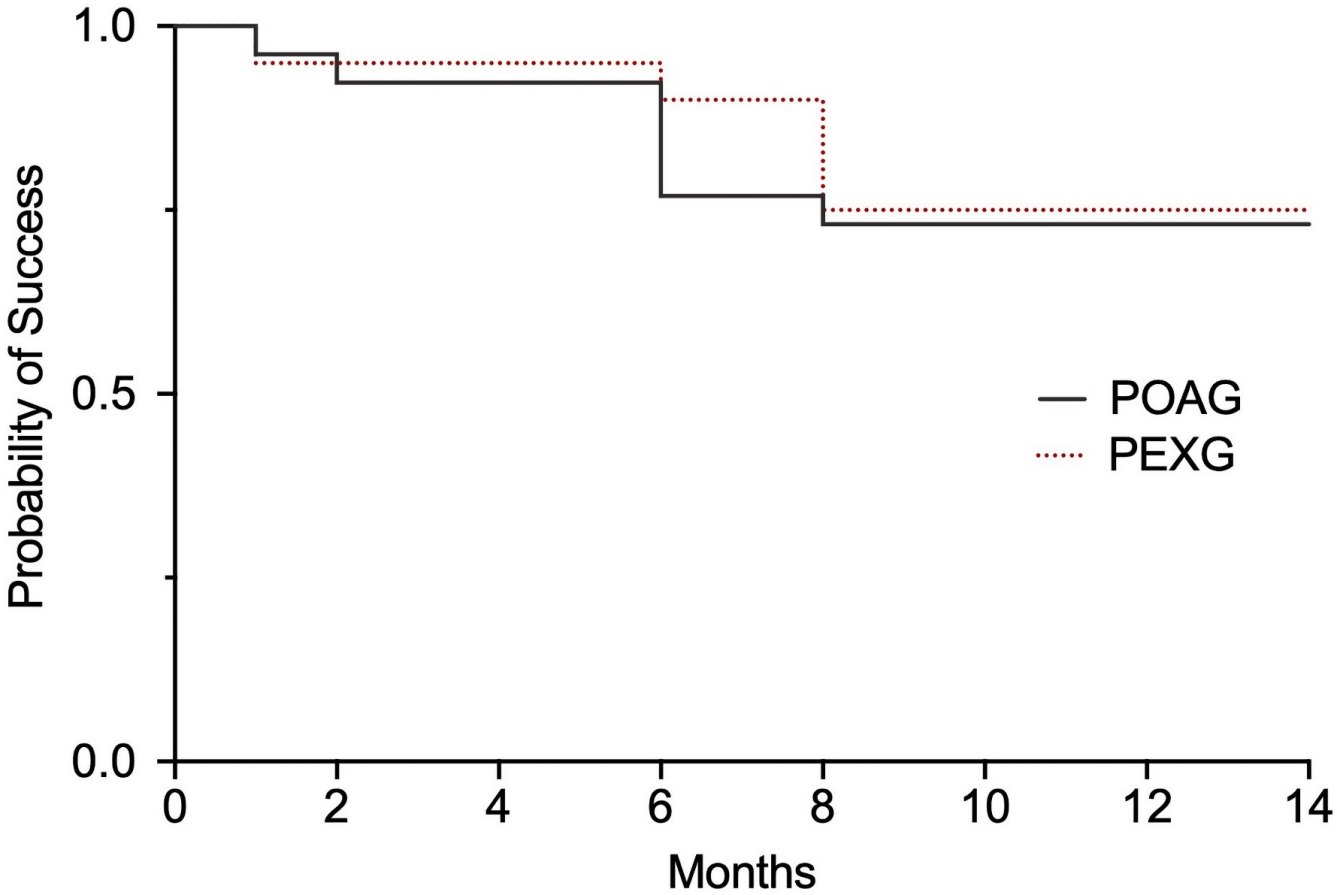
Kaplan-Meier survival curves. Kaplan-Meier survival curves for complete success. POAG = primary open angle glaucoma; PEXG = pseudoexfoliation glaucoma.

In the POAG group 73.1% (19/26) achieved complete success versus 75.0% (15/20) in the PEXG groups (*p* > 0.9999). Qualified success was achieved in 76.9% (20/26) in the POAG group versus 80.0% (16/20) in the PEXG group (*p* > 0.99999). Kaplan-Meier survival curves for complete success are depicted in Fig 5. One patient showed an IOP outside the range of 5 to 17mmHg at two consecutive visits in the POAG group (3.9%) versus none in the PEXG group. In the POAG group 5 patients had an IOP reduction from baseline of less than 20% at two consecutive visits (19.2%) versus one patient (5.0%) in the PEXG group. Further postoperative surgical interventions were necessary in 2 patients in the POAG group (2 surgical revision, 7.7%) versus 3 patients (2 surgical revision, 1 additional glaucoma surgery, 15.0%) in the PEXG group. The patient requiring additional glaucoma surgery received a cyclophotocoagulation. In each group 3 patients needed antiglaucomatous medication after surgery (11.5% versus 15.0%). There was no case of loss of light perception in both groups.

### Postoperative complications

Number and percentage of complications in each group are listed in Table 2. Two patients in the POAG group (7.7%) and 4 patients in the PEXG group (20.0%) had postoperative hyphema (*p* = 0.38). No action had to be taken due to spontaneous resorption. One patient in each group had a Seidel positive wound leak one month postoperatively (3.8% versus 5.0%). No case of bleb leak after one month post surgery was documented.

**Table 2:** Postoperative complications.

	POAG (n = 26)	PEXG (n = 20)	*p*
Hypotony [n (%)]	3 (11.5)	8 (40.0)	0.04
Choroidal detachment [n (%)]	1 (3.8)	6 (30.0)	0.03
Flat anterior chamber [n (%)]	3 (11.5)	3 (15.0)	> 0.9999
Macular folds [n (%)]	1 (3.8)	0 (0.0)	> 0.9999
Hyphema [n (%)]	2 (7.7)	4 (20.0)	0.38
Corneal complications [n (%)]	1 (3.8)	3 (15.0)	0.30
Corneal dellen [n (%)]	0 (0.0)	0 (0.0)	-
Corneal erosion [n (%)]	0 (0.0)	2 (10.0)	-
Corneal edema [n (%)]	1 (3.8)	1 (5.0)	-
Seidel positive [n (%)]	1 (3.8)	1 (5.0)	-
Implantat extrusion [n (%)]	0 (0.0)	0 (0.0)	-
Blebitis [n (%)]	0 (0.0)	0 (0.0)	-
Loss of light perception [n (%)]	0 (0.0)	0 (0.0)	-

n = number; *p* = p-value; PEXG = pseudoexfolation glaucoma; POAG = primary open angle glaucoma.

11.5% of the patients (n = 3) in the POAG group developed hypotony defined as IOP below 5mmHg versus 40.0% (n = 8) in the PEXG group (*p* = 0.04). Three patients in each group showed a flat anterior chamber (POAG 11,5% vs. PEXG 15.0%, *p* > 0,9999). Choroidal detachments were seen in one POAG patient versus 6 PEXG patients (3,8% versus 30,0%, *p* = 0.03). All cases of hypotony were seen immediately postoperatively, except for one case in the POAG group. In this case a pressure of 4mmHg was measured once in the time between three and four months postoperatively. Hypotony resolved spontaneously and IOP was 8mmHg at all further visits. All other cases of early hypotony resolved within 2 months postoperative. Spontaneous resolution of hypotony was documented in one POAG patient versus 4 PEXG patients, one case in the POAG group versus 2 in the PEXG group resolved with medical management and anterior chamber reformation using viscoelastics was necessary in one POAG patient versus 2 PEXG patients.

In both groups no vision threatening complication were documented. Furthermore, no case of corneal decompensation was seen at one year of follow-up.

### Postoperative interventions

Subconjunctival injection of 5-FU, needling, surgical revision and further glaucoma surgery were documented. In the POAG group subconjunctival injection of 5-FU was performed 37 times (1.42 injections per case) versus 25 times (1.25 injections per case) in the PEXG group. In 11 cases in the POAG groups injection of 5-FU was necessary only once (42.3%), 2 or 3 injections were performed in 10 cases (38.5%) and more than 3 injections had to be done only in one case (3.8%). 4 eyes (15.4%) did not get any injection postoperative at all. In contrast, in the PEXG group no injection was performed in 2 cases (10.0%), one in 11 cases (55.0%), 2 or 3 in 7 cases (35.0%) and more than three injections in no case. Total number of eyes needing 5-FU injections did not differ significantly between the two groups (*p* = 0.69).

Needling was either performed because of a flat bleb, a rise of IOP or a tenon cyst. Nine needling procedures (0.35 needling per case) were recorded in the POAG group versus 7 (0.35 needling per case) in the PEXG group. Total number of eyes undergoing needling did not differ significantly between the two groups (*p* = 0.54). Among POAG patients 17 cases did not need any needling at all, whereas 9 cases underwent needling once. No cases of two or more needlings were documented. Two cases of a tenon cyst were recorded, 7 eyes had an IOP rise and 5 showed a flat bleb. In the PEXG group needling was performed once in 3 cases and twice in 2 cases. Fifteen cases did not need any needling. In 3 cases needling had to be done because of an IOP rise and in 4 cases because of a flat bleb. One patient in the PEXG group developed a flat bleb because of blockage of the implant’s lumen through PEX material five months after surgery. Clearing of the lumen using Nd:YAG laser and consecutive needling was performed to restore implant function. IOP was controlled without any antiglaucomatous medication thereafter.

Surgical revision was considered as a failure. Two cases in each group were recorded (7.69% POAG vs. 10.0% PEXG). One patient in our cohort needed further glaucoma surgery to control IOP during the follow-up of 12 months. In this case, four months after initial, complicated surgery and surgical revision on the fourth postoperative day as well as unsuccessful needling cyclophotocoagulation was performed because of a scarred bleb.

## Discussion

This retrospective study of 46 eyes compared the efficacy and safety of microshunt implantation in POAG and PEXG. Subconjunctival microshunt implantation augmented with MMC was found to lower IOP effectively and reduce the number of antiglaucomatous medication in both groups. Complete success was achieved in 73.1% of patients with POAG and in 75.0% of patients with PEXG after 12 months of follow-up. Qualified success was accomplished in 76.9% and 80.0%, respectively. There was no statistical difference regarding surgical success rates. IOP was significantly lowered in both groups to the low teens. Furthermore, the number of antiglaucomatous medication was significantly reduced. At least 80% of the patients were completely off medication in each group after 12 months of follow-up.

Baseline demographic and clinical data showed significant differences between patients with POAG and PEXG. POAG patients in our study were on average 6.7 years younger than PEXG patients and mean baseline IOP was significantly higher in the PEXG group compared to POAG patients. It is known, that PEXG patients tend to be older and IOP fluctuations are higher as well as baseline IOP are higher at the time of diagnosis compared to POAG patients [4,5]. This is a known problem for studies comparing PEXG to other glaucoma entities, especially POAG. In several previous studies testing efficacy and safety of surgical procedures significant differences in age and mean IOP at baseline between PEXG and PAOG groups were documented [14–16]. Our study showed a significantly higher IOP reduction in the PEXG group leading to comparable IOP levels after one year of follow-up.

When analysing the postoperative complication rates within the two groups, a few differences were found. Rates of flat anterior chamber, macular folds, hyphema or corneal complications were comparable. Only cases of hypotony and choroidal detachment were distributed unequally. In PEXG patients such complications were significantly higher. Whether this discrepancy was caused due to PEXG specific disease characteristics or by the initial higher IOP in the PEXG group remains unclear. An aspect could be a different behaviour of the entry canal caused by alterations of scleral stiffness in the context of PEXG [17]. The canal may not close as well as in POAG, which would favour peritubular flow. A higher, sudden drop of IOP during surgery may favour the development of hypotony and choroidal detachment in the postoperative phase [18]. Nevertheless, hypotony resolved within 2 months postoperatively without any sequelae. No case of hypotony maculopathy or lasting visual loss was documented. Hypotony and choroidal detachment in previous studies regarding microshunt implantation were almost exclusively documented in POAG subjects and occurred in 13.0% to 39.0% and 2.0% to 12.9% of cases, respectively [12,13,19–21].

Needling rates in our study were the same in both groups, but higher than in previously published studies. Thus, needling rates of 5% to 19% were reported after on year of follow-up [20–22]. Comparison of needling rates is difficult, as there is no clear indication and as it is done on the decision of the treating surgeon.

Rates of complete and qualified success for both groups observed in our study are similar or even better compared to success rates of microshunt implantation reported in recent studies. For example, Schlenker et al. achieved complete success using very similar success criteria in 76.9% after one year of follow-up [13]. In another study with 12 months follow-up 58% and 79% achieved complete and qualified success, criteria being no IOP > 18mmHg at 2 consecutive visits after 3 months following surgery [21]. Durr et al. conducted a study on refractory glaucoma using similar success criteria finding a complete success rate of 61% and a qualified success rate of 79.9% one year postoperative [20]. In a large prospective, randomized, multicenter study comparing microshunt implantation and trabeculectomy in POAG, Baker et al. reported success rates of 65.1% in the microshunt group and 73.5% in the trabeculectomy group after one year of follow up when using definition of success proposed by the guidelines of the World Glaucoma Association [22]. Although comparison of success rates between studies is often compromised by using different success criteria, it gives a good impression on how to interpret the present results. Furthermore, we followed the guidelines of the World Glaucoma Association’s Guidelines on Design and Reporting of Glaucoma Surgical Trials.

Primary outcomes of surgical success rates were the same in POAG and PEXG eyes in our study. This result was not necessarily expected, as success rates after surgery, especially trabeculectomy, are inconsistent for PEXG. Lim et al. reported in their study similar results for PEXG and POAG eyes undergoing trabeculectomy augmented with MMC at one year of follow-up, but worse IOP control for PEXG from 2 years postoperatively [8]. In an additional study comparing trabeculectomy in PEXG and POAG without enhancement of antimetabolites complete success was significantly more common in POAG eyes [23]. Consequently, Li et al. found lower success rates for PEXG eyes compared to POAG eyes after 1, 3 and 5 years after trabeculectomy with MMC [24]. On the other hand, several previous studies reported comparable success rates up to two years of follow-up for PEXG and POAG after implantation of a XEN gel stent [15,16,25]. In accordance our study also showed similar results for microshunt implantation in PEXG and POAG. Therefore, minimally invasive glaucoma surgery with subconjunctival drainage seems to be a viable option in treating PEXG. Possibly, the minimally invasive approach does not exacerbate the breakdown of the already hampered blood-aqueous barrier in PEXG as much as standard glaucoma surgery, with consequently lower inflammatory cytokine levels in the anterior chamber and therefore lower rates of fibrosis and scarring [26]. Further research with direct comparison of trabeculectomy and minimally invasive subconjunctival drainage implants in PEXG is necessary to address this issue.

Using small lumen drainage implants, such as the microshunt, in PEXG has the disadvantage of possible outflow blockage through PEX material. We observed such a case in our study. To restore flow clearing the ostium using Nd:YAG laser is an easy, effective and safe option. If diagnosed early enough and there is still a prominent and functioning bleb, laser treatment alone might be successful. Longer lasting blockage will cause bleb degradation with scarring, making needling procedure unavoidable, still with high chance of restoring function completely.

### Limitations of the study

There are some limitations to our study. Because of its retrospective character the study population was not actively matched and therefore showed significant differences in patients demographics. PEXG patients were significantly older and had a significantly higher IOP at baseline, as mentioned before. Nevertheless, this setting reflects the everyday clinical routine and thus provides important insights into the efficiency and safety of the microshunt in everyday clinical practice. Another limitation of our study is the relatively small number of cases compared to other studies. Furthermore, the follow-up time is limited to 12 months and long-term efficacy and safety of microshunt implantation in PEXG are unclear. Finally, postoperative decisions to restart antiglaucomatous therapy or to perform surgical revision were not standardized and were at the discretion of the treating ophthalmologist.

## Conclusion

To the best of our knowledge, this is the first study comparing the efficacy and safety of microshunt implantation in POAG and PEXG with a follow-up of 12 months. Data of this study suggests that microshunt implantation is a safe procedure in PEXG and has similar IOP-lowering potential and surgical effectiveness in PEXG compared to POAG. Early postoperative hypotony and choroidal detachments seem to be more common in PEXG but resolved in all cases and were not vision threating.
